# A qualitative study of professionals’ perspectives on the ethics of medically-delivered safer injection education for people who inject drugs

**DOI:** 10.1186/s12910-023-00939-4

**Published:** 2023-08-11

**Authors:** Anastasia Demina, Caroline Desprès, Marie-France Mamzer

**Affiliations:** 1 Addiction medicine department, CHU Dijon Bourgogne, Dijon, France; 2 Université de Bourgogne, INSERM U1093 « Cognition, Action et Plasticité Sensorimotrice », Dijon, France; 3 Centre de Recherche des Cordeliers, Sorbonne Université, Université de Paris, Laboratoire ETREs, Inserm, Paris, F-75006 France; 4 Unité Fonctionnelle d’Ethique Médicale, Hôpital Necker-Enfants malades, APHP, 149 rue de Sèvres, Paris, 75015 France

**Keywords:** Harm reduction, IV drug use, Safer injection education

## Abstract

**Background:**

In this qualitative analysis we aimed to explore addiction physicians’ perspectives on safer injection education for people who inject drugs, especially: (1) on possible means of introducing safer injection education in the medical environment, (2) on the compatibility of safer injection education with each physician’s core values and goals, and (3) on possible reasons for the ethical dilemma in safer injection education.

**Methods:**

We conducted semi-structured interviews with eleven physicians practicing addiction medicine in France in clinical and harm reduction settings.

**Results:**

All participants were in favor of educational interventions for people who inject drugs. Nonetheless, these interventions varied from simple advice to injection supervision and they were seen as less acceptable when they concerned the practical and material aspects of injection. Some participants found that physicians practicing in clinical settings, where patients consult mostly to stop their drug use, should not practice safer injection education. On the contrary, other participants claimed that safer injection education was essential in all settings and was not a choice but rather a duty for addiction physicians. The ethical dilemma of such intervention when delivered by medical staff was viewed as a complex phenomenon, related to the representations of intravenous drug use and to societal expectations from physicians.

**Conclusion:**

Physicians’ views on safer injection education for people who inject drugs reveal an emotionally charged subject related to the structural organization of addiction management in France. Such education is marked by an arduous history of harm reduction policies in France.

**IRB registration:**

#00011928.

**Supplementary Information:**

The online version contains supplementary material available at 10.1186/s12910-023-00939-4.

## Introduction

Harm reduction (HR) is an essential part of addiction management in France. It is well known that drug injection carries significant health risks (viral transmission, bacterial infections, vascular complications, overdose, etc.) [1, 2] Also, the intravenous (IV) drug use is seen as a signature of a particularly severe addiction, and is often associated with significant socio-economic difficulties in people who inject drugs (PWID) [3].

Despite well-established evidence of the effectiveness of HR programs on various drug use complications, it remains at the centre of public and political discussions that follow new developments in HR in France [4]. The first HR approach in France consisted of needle and syringe programs (NSP), carried out under the sceptical gaze of medical professionals and public figures who claimed that removing risks from injection would contribute to the generalization of injection practices [5, 6]. In “Impossible prohibition”, Alexandre Marchant recalls that, in France, HR was a source of indignation among health professionals, politicians, and public opinion [7]. At the time, medical experts in addiction were primarily represented by psychiatrists with considerable psychoanalytical background and they were very reluctant to support such programs [6, 8]. The first opioid maintenance treatment (OMT) existed in a very limited, restricted and experimental setting, confronted with lively societal debate, demonstrating a deep apprehension of what was considered an overly laxist health policy on drug use. At that time, general practitioners practiced improvised substitution treatment to relieve their patients, which lead to regular criminal prosecution. Referred to as a “health and social catastrophe,” heroin addiction and the HIV epidemic forced policymakers to engage in HR. In March 1995, Methadone received its marketing authorization, formalizing the institutionalization of HR in France [7]. Despite the extremely cautious implementation - NSP being the only measure to accompany PWID for a long time, HR is now supported by legal texts in the Public Health Code, and is of the essence in modern addiction management in France [9]. It is, however, useful to note that in France there remains an institutional separation between healthcare facilities such as hospital wards, private practices, or Centres of Care and Prevention in Addiction medicine and HR facilities such as Support Centres for Harm Reduction for Drug Users and drug consumption facilities (DCFs).

Recent experimentation in DCFs is reviving old public debates [10, 11]. In France, DCFs mesmerize the public debate, centred on the repercussions of these facilities on the neighbourhoods in which they are located, despite the demonstrated positive effect of these structures on public drug use and delinquency [12]. DCFs and the new educational programs around injection HR could be a source of incomprehension and controversy affecting medical professionals who support such developments [10, 11]. Both society and peers may perceive the missions of medical professionals working in HR facilities as incompatible with their professional roles.

To explore these observations, we asked French addiction physicians to comment on their perceptions of safer injection education as part of injection HR and more particularly on their experience of teaching safer injection techniques for PWID. We aimed to explore their views on the acceptability of such measures and the adequacy with their professional roles in order to identify arguments that could justify or exclude medical practices related to safer intravenous drug use.

## Methods

This qualitative research was conducted by AD at the ETREs Laboratory (“Ethics, research, translations,“ University of Paris-Cité) and supervised by CD, a researcher in social and cultural anthropology and a public health physician. AD, herself an addiction physician with a Master’s degree in Ethics and Bioethics research, conducted exploratory bibliographical research to open up the questioning to sociological, anthropological, historical and political perspectives.

We conducted eleven semi-structured interviews with French addiction physicians. Participants were recruited by professional network and by snowball effect. To obtain a broad vision of professional perspectives, we chose to include professionals from different types of structures and settings, such as hospitals, private practices and HR structures. We did not formulate a limiting factor in the constitution of the sample in order to favour a wide range of expertise. We also decided to accept videoconference interviews to overcome schedule and geographical constraints. All appointments were taken by AD by telephone or email. One person refused to participate due to busy schedule constraints. The interviews took place at the participants’ workplaces.

Our interview guide consisted of open-ended questions on injection HR. The framework of the interview guide was adaptable to the context. We deliberately did not include theoretical questions and we were careful not to ask questions that could induce answers. The interview guide was pilot tested in March 2022. The final version of the interview guide is presented in the Appendix.

For our qualitative analysis, we used the Framework Analysis method [13]. After interviews, transcriptions, and thorough familiarization with transcribed data, we proceeded to a manual coding of all transcribed interviews. We avoided purely descriptive coding, trying instead to express every interviewee’s individual experience. Once the coding was completed, we looked to organize the identified codes into categories. We integrated articulated categories by studying their variability in various contexts to analyse our data as a whole and to build the narrative synthesis of our findings. No software was used to manage the data.

For the preparation of the manuscript, we adhered to the Guidance for reporting qualitative manuscripts [14].

### Consent, information, ethics

Our information note described our research broadly in order to avoid over-preparation and thought induction. At the beginning of each interview, the physician was reminded of the recording and was asked to confirm his/her informed consent. All interviews ended with the collection of the participants’ general impressions. AD used a recording device and took notes by hand to collect non-verbal information. The interviews were entirely transcribed by AD, with exact words and phrases used by the participants and remaining faithful to their intonations and emotional expressions. Thus, all perceived verbal and non-verbal information was gathered for each interview and analyzed.

Video interviews took place on an encrypted platform. The recordings were erased once the interview was transcribed. No identifying information was transcribed. There is no document linking the interview number to the interviewee. Given the sensitivity of the subject, we chose not to make the transcripts public. Transcripts were saved on a secure digital device and were analysed internally in the ETREs laboratory. The raw data will be erased in 5 years.

We filed the data processing declaration with the University Paris-Cité Data Protection Officer. The Assistance Publique – Hôpitaux de Paris Ethics Committee granted approval for this study (IRB registration: #00011928).

## Results

### Description of the sample

Our eleven interviews were each approximately 30 to 50 min long, with an average duration of 38 min. The interviews were conducted face-to-face for three participants, by videoconference for seven participants and by phone for one participant, at his request. All interviews took place in France.

Among the physicians interviewed, there were five women and six men. Ages were censored for confidentiality purposes. In our sample, five doctors had psychiatric training, four were general practitioners, and two represented other specialties.^1^ Two nurses participated in this study at the request of one physician. Their experience was also transcribed and included in our analysis. Two participants worked in specialized HR settings; all other participants mostly intervened in healthcare structures. General characteristics of the interviewees are presented in Table 1.

**Table 1:** Participant characteristics, fields of practice, link to Injection HR and acceptable injection HR procedures

Interview	Initial training	Place	Field of practice	Link to the research question
I1	GP	hospital	General addiction medicine including PWID	No direct link to injection HR. Support delegated to HR structures. Possible means: general advice. Main argument: does not feel competent enough in this matter, the structure is not adapted to injection HR and the demands of the patients are for maintaining abstinence and not for HR.
I2	psychiatry	hospital	Complex follow-ups, expert consultations	Link: important link to injection HR (career, research). Practical measures of injection HR impossible in a health structure. Support delegated to HR structures. Argument: “how far could it go”
I3	other specialty	HR structure	General addiction medicine including PWID	Link: works in an HR structure. Injection HR seen as essential. Possible means: through discussion, explanations, veinous access research, explanations concerning the paraphernalia. No real-time injection supervision. Highlights the lack of time in a medical schedule precluding the possibility of injection HR.
I4	psychiatry	hospital	General addiction medicine including PWID	Link: worked in HR in carceral setting. Injection HR only possible if in connection with a therapeutic objective for another substance; no possibility of a medical follow-up exclusively for injection HR. Possible means: general advice and information, practical advice on the use of the paraphernalia.
I5	GP	HR structure	General addiction medicine including PWID	Link: important link to injection HR (career, research). Injection HR seen as essential. Possible means: general advice and information, practical advice on the use of the paraphernalia, use of an arm model in group setting, information on the preparation of the substance.
I6	other specialty	hospital	General addiction medicine including PWID, expert consultations	Link: associative work, link with HR in the context of expert consultations. Finds injection HR essential. Possible means: general advice and information, practical advice on the use of the paraphernalia. Argument: the opposite is hypocritical; one cannot be an addictologist without being an HR specialist.
I7	GP	private practice	General addiction medicine including PWID	Link: practices injection HR. Possible means: real-time injection supervision. Argument: personal story of meeting a user, utility, relationship.
I8	GP	hospital	General addiction medicine including PWID	Link: important link with injection HR (career). Possible means: general advice, information about safe injection tutorials, screening tutorials. Argument: respect for the person, utility, relationship.
I9	psychiatry	hospital	Complex consultations, expert consultations	Link: direct link to injection HR (research). Support delegated to HR structures. Argument: organizational constraints (doctors should engage in purely medical activities).
I10	psychiatry	hospital	Complex consultations, expert consultations	No direct link to injection HR. Injection HR delegated to HR structures. Argument: others do it as well and engage fewer resources. No fundamental moral tension with injection HR.
I11	psychiatry	hospital	Complex consultations, expert consultations	No direct link to injection HR. Possible means: general advice, information about safe injection tutorials.

GP – general practitioner, PWID – people who inject drugs, HR – Harm reduction

### Dual perception of HR

To understand participants’ perspectives on safer injection education for PWID, we first explored their views on HR in general. Some participants understood by HR any action aimed to reduce the physical and psychosocial consequences of drug use. In this interpretation, HR was integrated into medical care. The second interpretation was less frequent and opposed HR and medical care; HR being interpreted as pragmatic and mostly consisting of sterile material distribution, unrelated to medical care. The ways of conceptualizing HR seemed to be related to professional practices. The first, more global vision of HR was prevalent in our sample, being adopted by nine participants, mostly general practitioners.

The distinction between the two interpretations of HR may be related to the institutional separation between HR structures and healthcare structures in France. This separation is often critically discussed by the actors in addiction medicine. In fact, in our sample some interviewees regretted it:

*“… and what I regret in our approach to these questions, uh… it’s this kind of fragmentation… it’s this kind of separation between care uh… real medical care – the one which is really care because in France care is always medical… and what we would call … social support or help for survival…” (I5)*.

Other practitioners found this separation meaningful and important in meeting the demands of those who seek abstinence:

*“Everyone understands that HR must be everywhere – it’s true… But some patients ask for help… to maintain abstinence… and … despite everything, we should also have places uh… that are different… uh… places oriented towards abstinence in which HR cannot enter…” (I9)*.

### Perceptions of safe injection education

In our study, all physicians were in favour of safer injection educational interventions. However, we noted a rather ambiguous relationship to such interventions when discussing the physician’s role in this injection education.

The interviewees shared their experiences of different varieties of safer injection education. Some proposed general advice during medical consultations on sterile material. Some proposed more practical advice:

*“I mean, we have to stop the hypocrisy – we give them sterile equipment! So (laughs) we’re not going to say hum… I don’t want to know how you inject and I’m not going to teach you how to inject well, but please take clean syringes…” (I6)*.

Some used educational materials during group workshops. For one practitioner, it was a video sequence of safe injection. For another, it was an artificial arm:

*“… you know, plastic arms – that can be a pretty good workshop to see how they plant the needle, uh… how they manage… the inclination of the needle…”(I5)*.

These workshops were mainly designed for people seeking HR in specialised structures, and as for healthcare facilities, this type of workshop seemed inconceivable for some participants:

*“But the HR of injection … we can do it for very precarious patients that we see individually, that of course, but… We don’t institutionalize that in terms of… patient counselling and support groups.”(I2)*.

As for the real-time injection practice, opinions differed as well. Certain professionals helped PWID to find a venous access. They could also supervise the injection of saline solution, or the drug injection itself. For most of the interviewees, this practice was unacceptable, especially in healthcare facilities:

*“We’re not going to help with injection… We can say to ourselves… yes but…if we engage in that, it can go very far… So, I think that… we, doctors uh… and nurses of the sanitary uh… we must be in the care process…” (I2)*.

### Should physicians educate patients and users on safe injection? Practical and ethical arguments

Participants considered this question from different perspectives. Indeed, some considered physicians less competent in safer injection education than nurses who have more extensive training in injection technique. Some reported the difficulty of including such time-consuming educational support in their busy medical schedules.

On the other hand, safe injection educational support was justified by the practitioners’ awareness of the risks of injection, particularly when it is performed in an inappropriate way. Participants described injection as a complex gesture, requiring training even for health professionals. Some outlined observations of poor knowledge of anatomy in PWID, resulting in dangerous injection techniques.

As for the ethical arguments, some participants evoked the physician’s mission to prevent painful and mutilating injection complications. In this case, safer injection education was considered as a duty (their responsibility, their “job” as they put it), not a choice.

Several practitioners also formulated the goal of “bringing the person into care” as an essential component of injection HR and safer injection education. Therefore, safer injection education was considered temporary, as a first step, while waiting for a “real” therapeutic goal of abstinence. Other practitioners critically discussed this perspective. They advocated an attitude of acceptance of continued drug use as a personal choice, with non-judgmental valorisation of the person’s capacity to decide in line with their values. For these participants, HR was an adequate response to the reality of drug use, requiring a detachment from questions of morality, questions of good or evil.

*“I’m not going to judge… I can’t judge people’s choices and behaviours - that’s related to the prohibitionist system and all that - it’s a practice that’s about the individuals themselves…” (I5)*.

Some participants saw the safer injection educational support on the part of medical professionals as humanistic, compassionate, and respectful.

*“It’s (long pause, thinks) it’s the story of respect…- it’s the story of…that the person knows that…uh…the caregiver is not there to criticize them, it’s just…it strengthens the bond…” (I7)*.

There seemed to be a fundamental contradiction between those who found that physicians have their role to play in safer injection education and those who reserved such interventions to non-medical staff. Deontological considerations were invoked in both visions: those who accepted educating PWID on safer injection considered it their most evident duty as a physician, a duty which has an immense practical value in preventing injection complications. However, it was the duty of “curing” PWID, reaching for the “ideal of abstinence” that was prioritized by the physicians who preferred to leave safer injection education to non-medical professionals.

### Social representations concerning injection and medical professionals

Overall, safer injection education seemed acceptable for our participants. Nevertheless, the more this education approached real-life injection, the less acceptable it appeared to them, especially in healthcare settings. It seemed that for some participants moral tensions arose because of the dominant social representations of drug injection in society, at least partly internalized by these professionals. In France, professionals and policymakers were very cautious with HR measures which resulted in a significant delay of French HR implementation compared to other European countries. The moral interpretation of this delay, attributing it to the social representation of the injectable drug use as a “moral vice”, is predominant for French social studies scientists [2, 15].

First, there was the very nature of injection, seen by two of our interviewees as “shocking,“ “dirty,“ “violent,“ “morbid” and “deadly,“^2^ with the ideas of contamination through the violent intrusion of the skin barriers:

*“These topics are so controversial (gesticulates)… so disturbing… It’s so debated… like… like… harm reduction… drug consumption rooms… so… well… It’s so dirty (gesticulates)… It’s something very serious…” (I2)*.

*“I tend to think that injection is a relatively morbid act. And uh… and yeah… the guy, he’s practically taking a blood test! He’s injecting himself with something… he’s putting the needle, he prepares the thing, he injects a drug into his body! If we put that on paper, it’s… it’s super violent in fact… It’s extremely violent…” (I6)*.

The sensual aspects of injection were sometimes discernible in interviewees’ responses, referring to hetero-injections and to the needle penetration. As for the psychoactive effect, vocabulary like “extreme high,“ “ecstasy” or “orgasmic sensation,“ going beyond clinical experience and referring to an almost fantasized imagination, was often used. Our participants also evoked a demonized side of injection, source of exclusion and secrecy, with IV drug use viewed as a scandalous or “taboo” practice:

*“At the beginning of the last century, uh where clearly injections of opiates were the archetype of immoral and decadent use… uh… At the time there were essentially subcutaneous injections uh… in the thighs… And it was a part of the body that couldn’t be shown at all… uh… so there is something like that, a bit scandalous…” (I4)*.

Some participants evoked the existing contradiction between injection education and addiction specialists’ historical mission in France, psychoanalytically oriented, seeking abstinence. As for the physician’s role and societal expectations concerning the physician, also possibly internalized by the caregivers, injection education appeared as possibly dissonant. There are societal requirements for physician’s moral and behavioural exemplarity, as described in French Medical Deontology Code. In one of the opening chapters, in addition to the duties of respect for human life and dignity, it is stated that, “the doctor must, in all circumstances, respect the principles of morality, probity and devotion essential to the exercise of medicine” [16]. In commentary to this legal document included to the French Public Health Code, morality is defined as referring to the societal norms and to the laws of the democratic society. It is worth reminding that, in 2016, the National Council of the Order of Doctors, the authority ensuring French physicians’ compliance with medical ethics and deontology, was opposed to the opening of first French DCF [2].

Professional and social representations of drug injection seem to influence controversies on safer injection education. In face of such representations, imagining a physician teaching safe injection to PWID could revive emotionally charged debates on drug use, HR and professional integrity.

## Discussion

The question of the compatibility of safer injection education with the responsibilities of physicians oriented the empirical part of this study. This qualitative study does not attempt to generalize or to measure a prevalence of representations and attitudes. It aims to describe different forms of safer injection educational practices, to clarify some of the professional positions, and to identify possible sources of ethical tensions.

There was no fundamental refusal of this practice in our interviewees’ discourses. Certain forms of reluctance were nuanced in terms of acceptable ways to perform injection education, particularly in relation to the type of structures in which it took place. It seemed that the attitudes around injection were at least partly influenced by the professional representations of injection marked both by disgust and fascination around this gesture, perceived as profoundly deviant, forbidden, but at the same time resulting in an extreme effect described in strongly sensual terms. This ambivalent association with the scandalous and the fascinating calls to mind the early history of recreational morphine injection. Opiates were consumed subcutaneously for recreational purposes from the mid-19th century, when the need to manage pain on the battlefield and the discovery of morphine in 1804 catalysed the manufacture of the first modern syringe [17–19]. “Soldier’s disease” rapidly extended to worldly social circles, in which morphine injection became ambivalently associated with pleasure and decadence, with a strongly scandalous and sensual imprint, given that women often injected in their thighs, at a time when even exposing one’s ankle was considered outrageous [17]. Most participants critically discussed this imprint of the imaginary on medical practice, preferring to distance themselves from it.

Different forms of safer injection education were proposed by our participants, forming a spectrum of possibilities. It seems that there is a separation between what could be considered as therapeutic education (general advice) and other interventions which are more related to the reality of injection, which are not considered therapeutic, and which are often delegated to other professionals or to peers. This finding could be interpreted as the idea of the impossibility of medical participation in drug injection, an obviously harmful behaviour medically speaking. This idea was criticized by most of our participants, who see in injection HR a therapeutic promise and the possibility of building a therapeutic relationship, in addition to its obvious preventive value.

The dual understanding of HR occupied a major place in our interviewees’ discourses. This duality may be related to historical and cultural aspects specific to HR in France, and it is reflected in the institutionalization of HR outside of healthcare facilities. As it was conceived in France, HR remains separate from healthcare, seemingly establishing a barrier between users and those who are abstinent.

This institutional separation between healthcare and HR may favour the exclusion of PWID from health facilities, and thus precluding those in need access to patient-centred care [20]. To be able to access a healthcare structure, even for a non-addiction-related issue, the user must agree to abstinence, most of the times confirmed biochemically with screening tests. In this perspective, those who continue their IV use could be seen as Michel Foucault’s “abnormal”^3^ who must be pathologized with an objective of control over what is considered a social danger, a public disorder, or a deviance [21]. Nevertheless, it is useful to remember that deviance and the abnormal are not immutable data from natural laws, but a societal construction, separating “healthy” practices from “deviant” practices, sometimes for control and dominance purposes [22].

PWID remain separated from those who have “repented” by accepting abstinence. This institutional fragmentation could be seen as counterintuitive given the complexity of users’ “careers.” The dynamic aspect of a user’s “career,“ as defined by Howard Becker in Outsiders, is translated into a vision of sequential evolution in a user’s practice [22]. Thus, PWID are not “condemned” to remain IV users forever, for there exists the possibility for an evolution from this step of user’s “career” to another, and it is worth considering the risk of blocking PWID in their IV use with this institutional separation, increasing their social isolation and vulnerability.

Treating chronic disease includes a mission of support, aimed at limiting the consequences of the disease and “controlling the disorders it causes,” a responsibility seemingly fully in line with HR [23]. Public health indicators establish its practical usefulness [24]. Yet, despite these justifications, injection HR seems to remain disturbing. It may seem that safer injection education represents HR pushed to its extreme, and medical participation in this process could be perceived as problematic. Indeed, it could be seen as symbolical or practical participation in a “deviant” process which is completely disconnected from medical care, and even as endorsing the continued IV drug use. Yet, qualitative literature regarding French public opinion on injection HR in DCF seems to indicate that their acceptability increased if health professionals ran these structures and actively encouraged PWID to stop their use [2].

The separation between healthcare and HR is thought to be highly operative and safer injection education seems to have its place only when restricted to HR facilities. Thus, given the extremely rare medical presence in HR facilities, physicians are mostly absent from injection HR. As a result, people who continue to use drugs are symbolically denied medical attention and expertise. This situation may reflect the common belief that in order to engage on a path to better health, it is necessary to stop using drugs completely. Thinking of drug use solely in terms of disease and medical complications not only completely obscures the cultural and societal dimensions of drug use, but also leads to a certain “medicocentrism,“ bypassing those aspects that are not considered purely medical such as housing, finances or even survival. Conversely, addiction physicians may be seen as actors of social control on drug use by at least some users, and therefore they might not be the most “legitimate” professionals to intervene in injection education.

Our study has limitations. The first limitation is due to our sample size, although given our research question and the exploratory nature of our qualitative study, this limitation does not seem to preclude our analysis. In addition, we explored only the perceptions of medical professionals in the medical field of addiction. To complete this analysis, it is crucial to explore the perceptions of PWID. Thus, future qualitative research is needed to better understand PWID’s understanding of the role of medical professionals in safer injection education.

We accepted video interviews due to the schedule constraints for our participants. It is possible that the video interviews precluded a thorough transcription of the non-verbal information. Nevertheless, in context of the COVID pandemic, the participants were used to work on encrypted video conference platforms and some of them largely preferred video interviews. In addition, we were interested in a perspective from professionals with diverse backgrounds working in different structures in France; therefore, video interviews were an acceptable option to overcome geographical constraints.

Another limitation is related to the first author’s direct relation to the explored field of addiction medicine, although a concerted effort was made to broaden her perspective on the research question. To minimize potential bias related to her professional background, she distanced herself from her own professional understanding of the injection HR. She attempted to interview from a neutral position, without passing judgement. For this, her research supervisor, and other researchers of the ETRES lab critically discussed her research progress from anthropological, sociological, philosophical and medico-economical points of view during regularly organized meetings.

To imagine future developments in HR, a change of perspective on drug use seems essential. The failure of the “war on drugs,“ resulting not only in colossal expense without significant effectiveness, but also generating incarcerations, racial inequalities and police violence, is widely established [25].

Some scientists argue that HR is a political or cultural issue rather than medical one. In our study, some interviewees argued that the most prominent in HR countries shared cultural features which favored a more pragmatic rather than moral perspective on HR. Studies in French general population, although limited by the convenience sampling, indicate that the acceptability of DCFs in France depended on interviewee’s political orientation with more conservative interviewees having more difficulties in accepting DCFs [2]. A certain antagonism to the HR from conservatively oriented political actors was observed in Canada in 2007 [26]. Furthermore, some researchers claim that cultural and historical contexts are of importance for understanding HR from an international perspective. For instance, Des Jarlais et al. attributes the difficulties in HR implementation in the United States of America to a strong Puritan imprint in civil laws, and a demonization of drug use historically associated with stigmatized racial minorities [27]. In Russia, HR is not endorsed by the government, OMT is illegal, drug users are incarcerated, and the prevalence of HIV exceeds 1% of the general population [28, 29].

In today’s France, valuing diversity, vulnerability and interdependence, the dysfunctions of the repressive system are recognized [30]. It is essential to continue identifying sources of exclusion in order to rethink our practices and create different drug regulation policies, favouring cultural sensibility and general access to patient-centred care for PWID.

### Electronic supplementary material

Below is the link to the electronic supplementary material.

Additional File 1: Interview Guide

## Data Availability

Data sharing is not applicable to this article as no datasets were generated or analysed during the current study.

## Footnotes

1 In France, addiction medicine or addictology is a medical sub-specialty. Therefore, all addiction physicians in France have training in different specialties such as general medicine, psychiatry, etc., in addition to their sub-specialty.

2 Expressions used by the participants.

3 Michel Foucault in his College de France lectures “The abnormal” proposed his understanding of historical mission of psychiatry in France: for him, it was a mission of social protection from the consequences of mental disorders, a “public hygiene” mission, put into practice through pathologization of social dangers.

## Declarations

### Registration details

IRB registration: #00011928.

## Abbreviations

HR: Harm reduction
IV: intravenous
PWID: people who inject drugs
NSP: needle and syringe programs
OMT: opioid maintenance treatment
DCF: drug consumption facility
