# Supplementary material for: A qualitative study of professionals’ perspectives on the ethics of medically-delivered safer injection education for people who inject drugs
Source: BMC Med Ethics. 2023 Aug 11;24:63. doi: 10.1186/s12910-023-00939-4 (PMC10422818; doi:10.1186/s12910-023-00939-4)
Supplement: Supplementary file 1 — Additional File 1: Interview Guide [file 12910_2023_939_MOESM1_ESM.docx]

**Interview Guide**

**Part One: The patients**

***Could you please tell me about your practice as an addiction specialist?***

***What patients do you encounter in your practice?***

***Among these different profiles: are there profiles that are particularly difficult for you?***

***-*** *If mentions IV drug use: why is it different from other types of use?* ***-*** *Otherwise: Do you practice HR?*

**Part Two: Physician interacting with patients**

***How do you see your job, your role, your mission? What is the purpose of the care you provide?***

*- And HR, what do you think about it?*

***What are the main difficulties you face in practicing HR?***

**Part Three: Safe injection educational support for PWID**

*What does injection in the context of IV drug use evoke for you?*

***Have you ever taught another person how to inject (colleague, patient, user)?****What is different in these learning processes? (see if differentiation made between health and recreational goals of injection)*

***What does it mean to you, the fact that a person is consuming substances by injecting them?***

*If the physician practices safe injection support:* ***Can you describe a typical session? What are your difficulties? What does it change for the user? Or for you?****Do you do other HR interventions? Why are they different?*

***Imagine the situation of a person asking you for advice for a first-time injection, so it is a person who has never injected. What would your attitude be? How is it different?***

***Why do you practice injection HR? Or why don't you?***
